# Polymer-Templated Durable and Hydrophobic Nanostructures for Hydrogen Gas Sensing Applications

**DOI:** 10.3390/polym13244470

**Published:** 2021-12-20

**Authors:** Mohammad Kamal Hossain, Qasem Ahmed Drmosh

**Affiliations:** 1 Interdisciplinary Research Center for Renewable Energy and Power Systems (IRC-REPS), Research Institute, King Fahd University of Petroleum & Minerals (KFUPM), Dhahran 31261, Saudi Arabia; 2 Interdisciplinary Research Center for Hydrogen and Energy Storage (IRC-HES), Research Institute, King Fahd University of Petroleum & Minerals (KFUPM), Dhahran 31261, Saudi Arabia; drmosh@kfupm.edu.sa

**Keywords:** polymer, hydrogen, hydrophobic, sensing, nanostructures, palladium

## Abstract

A simple and hands-on one-step process has been implemented to fabricate polymer-templated hydrophobic nanostructures as hydrogen gas sensing platforms. Topographic measurements have confirmed irregular hills and dips of various dimensions that are responsible for creating air bubble pockets that satisfy the Cassie–Baxter state of hydrophobicity. High-resolution field-emission scanning electron microscopy (FESEM) has revealed double-layer structures consisting of fine microscopic flower-like structures of nanoscale petals on the top of base nanostructures. Wetting contact angle (WCA) measurements further revealed the contact angle to be ~142.0° ± 10.0°. Such hydrophobic nanostructures were expected to provide a platform for gas-sensing materials of a higher surface area. From this direction, a very thin layer of palladium, ca. 100 nm of thickness, was sputtered. Thereafter, further topographic and WCA measurements were carried out. FESEM micrographs revealed that microscopic flower-like structures of nanoscale petals remained intact. A sessile drop test reconfirmed a WCA of as high as ~130.0° ± 10.0°. Due to the inherent features of hydrophobic nanostructures, a wider surface area was expected that can be useful for higher target gas adsorption sites. In this context, a customized sensing facility was set up, and H_2_ gas sensing performance was carried out. The surface nanostructures were found to be very stable and durable over the course of a year and beyond. A polymer-based hydrophobic gas-sensing platform as investigated in this study will play a dual role in hydrophobicity as well as superior gas-sensing characteristics.

## 1. Introduction

Gas sensing, particularly hydrogen (H_2_) gas sensing, has become very crucial due to its renascence as a new and alternative energy in modern life [1,2,3]. H_2_ is an important energy carrier that is going to be complementary to current electricity very soon [4,5]. A persistence challenge is being carried out to incorporate H_2_ as fuel for “zero-emissions” vehicles, to heat accommodations and workplaces and fuel aircrafts, amongst many other applications [6,7,8]. Therefore, not only is an efficient and sensitive sensing platform urgently needed for the safe deployment of all H_2_-based applications, but also multifunctional capabilities are required to deal with extreme and critical environmental conditions. Most H_2_ gas sensor*s*, particularly used in industries and workplaces, are not suitable for advanced and sophisticated applications. The sensor needs to be smart, durable and of multitasking capacity [9,10,11]. The fabrication and realization of multifunctional sensing platforms have been exciting and hot areas of research in both academia and industry, including healthcare sectors and environmental protection [12,13]. There is an enticing and ever-growing interest in devising a sensing platform capable of hydrophobic and non-adhesive characteristics [14,15,16]. Such platforms facilitate water droplets dropped onto them, rolling them off automatically with a small tilt angle. In the process of rolling off, contaminants and dust are also carried away, and thus the sensing surfaces become ready to detect the target gas [17,18]. However, developing an artificial hydrophobic sensing surface is not that straightforward, and it requires a smooth and fine strategy so that the surface becomes stable, durable and capable of reconciling with the surrounding environment [19,20,21,22]. Most of the time, a hydrophobic surface is achieved by following the improvised Cassie–Baxter model that confirms a double-layer roughness wherein there should be nanoscale roughness on the top of the microscale structures [23,24,25]. Although such double-layer structures have great potential in a wide range of applications, including self-cleaning, anti-fouling, anti-corrosion and oil–water separation, the top nanostructures decay over time under extreme environmental conditions [26,27].

A wide range of organic and inorganic materials are used as base substrates to achieve hydrophobic sensing surfaces [28,29,30]. Polymers, particularly polycarbonate (bisphenol A polycarbonate: 2,2-bis(p-hydroxyphenyl)-propane, PC), are one of the interesting base materials that have been studied extensively and therefore used in a wide variety of applications [31,32,33,34]. Due to low-cost, high durability, low modulus of elasticity and high transparency, PC has been useful in optoelectronic and microelectronic applications [35,36,37]. PC is an industrially attractive soft polymer material that is being extracted as a by-product from oil and gas refineries through industrial-scale and low-cost processes [33,38]. However, one finds that such PC can be further reinforced by including hydrophobic characteristics, which are a key element in many applications that require a self-cleaning property. It is well-acknowledged that the effectiveness of hydrophobicity is demonstrated by the Cassie–Baxter model, although the same model indicates that a higher surface area is required for many surface-enhanced applications, such as molecule detection and sensing [39,40,41]. For multifunctional devices, particularly hydrophobic gas-sensing platforms, using PC as the base materials is industrially viable and one of the promising candidates. To the best of our knowledge, a PC-templated hydrophobic gas-sensing platform has not been reported so far. Mazen and his group have demonstrated and devised a transparent hydrophobic PC as a self-cleaning surface for PV panels installed in a dusty environment [42]. Yilbas and his team have developed a generic process to copy the micro/nanoscale structure of hydrophobic PC by polydimethylsiloxane (PDMS) that showed a higher wetting contact angle (WCA) and transparency [43]. Jhang and his group developed hydrophobic microchannels in PC that enabled the valve-free sequential injection of multiple liquids [44]. Most of the methods used in achieving such hydrophobic PC were associated with multiple treatments, apart from the requirement of skilled hands and the usage of specialized reagents.

Here in this study, we have reported a simple and inexpensive one-step process to fabricate polymer-templated hydrophobic nanostructures for a H_2_ sensing application. A hydrophobic PC fabricated by a wet chemical treatment was decorated with palladium (Pd) by high vacuum sputtering technique. High-resolution field-emission scanning electron microscopy (FESEM) revealed double-layer structures consisting of fine microscopic flower-like structures of nanoscale petals on the top of base nanostructures. Sessile drop tests confirmed a WCA of the treated PC as high as 142 ± 10.0°, whereas the WCA of pristine PC was estimated to be ~83 ± 10.0°. The as-fabricated polymer-based nanostructures were transferred to an automatic sputtering chamber for Pd sputtering and decorated the treated PC with Pd. Sessile drop tests were carried out once again to evaluate the WCA. It was noted that the Pd-decorated treated PC exhibited a relatively high WCA of ~130 ± 10.0°. A customized setup was built to carry out the sensing characteristics of the Pd-decorated treated PC. Such a generic strategy is indispensable to explore new routes of multifunctional sensing platforms that are particularly important in extreme environmental conditions.

## 2. Materials and Methods

**Figure 1:**
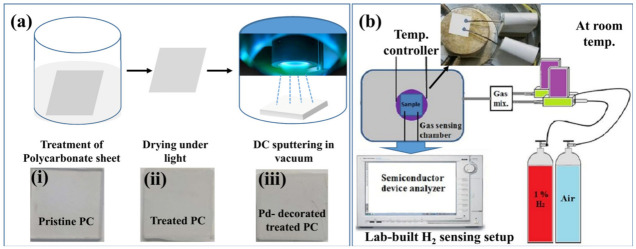
(**a**) A free-hand schematic for fabricating a multifunctional sensing platform; inset (**i**–**iii**): CCD images of pristine PC, treated PC and Pd-decorated treated PC; (**b**) customized H_2_-sensing setup used in this study.

Commercially available PC sheet (1 mm × 1 mm × 1.6 mm) was cut into pieces (2.5 cm × 2.5 cm × 1.6 mm) and treated with 2-propanone (CH_3_–CO–CH_3_) under controlled lab conditions. The sample was washed copiously with deionized (DI) water. A transparent pristine PC specimen was found to turn opaque after the treatment. The treated PC was left under a fluorescent light of 30 W for 10 min. and then transferred to an automatic sputtering coater (model #NSC 4000; NANO-MASTER Inc., Austin, TX, USA) for Pd decoration, as shown in Figure 1. Inset (i)–(iii) of Figure 1a represents CCD images of pristine PC, treated PC and Pd-decorated treated PC. High purity Pd (99.999%) target was purchased from Semiconductor Wafer Inc. and used as received without any modification. Pre-sputtering for cleaning the target was carried out for 1 min. Plasma was generated by direct current (DC) magnetron power of 30 W for 20 s, keeping the chamber background pressure as low as 3.5 × 10^−6^ Torr in Ar gas flow of 80 SCCM. The target-to-substrate distance was fixed at 10 cm.

Initial assessment of surface topography and roughness was carried out by using a 3D optical microscope (model #Meiji Techno MX7100; Meiji, IL, USA). A Dektak profilometer (mode #BrukerXT; Bruker, MA, USA) was used to explore the microscopic surface structure of the treated specimen. Topographic confirmation and in-depth morphology of treated specimens were carried out using high-resolution FESEM (model #LYRA3; TESCAN, Brno, Czech Republic). WCA measurements were carried out using a goniometer (model #DM 501; Kyowa Interface Science Co. Ltd., Saitama, Japan) through sessile drop tests. DI water was used in the sessile drop experiments, and the droplet volume was controlled with an automatic dispensing system. The images of the droplets were taken one second after deposition of the water droplet on the surface. A customized gas chamber, Linkam stage (Model HFS-600E-PB4; Linkam Scientific Instruments, Wakefield, UK), was used to incorporate air and H_2_-balanced nitrogen (1% H_2_, 99% N_2_) sequentially at room temperature. Two mass flow controllers (MFCs) connected with an external XPH-100 power hub supply were utilized to control the flow of H_2_. The sensing measurements were performed through a resistivity measurement approach at room temperature. It is well-acknowledged that resistivity defines the ability of the materials to resist the charge flow, whereas the charge carriers available in the vicinity depend on the adsorption of target gas molecules on sensing materials.

## 3. Results and Discussion

**Figure 2:**
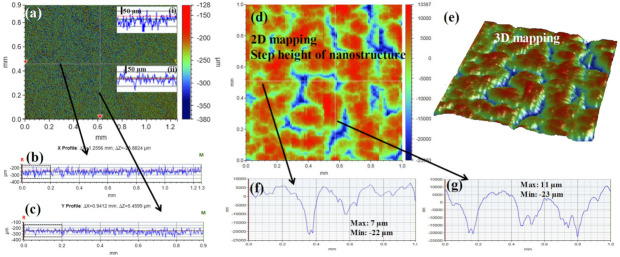
(**a**) Large area 3D optical microscopic image of treated PC; (**b**,**c**) line profiles along the horizontal and vertical axis as marked by black arrows in Figure 2a, respectively; (**d**) 2D image of the same extracted from surface profilometer; (**e**) 3D mapping of the same area confirming hills and dips; (**f**,**g**) line profiles along the horizontal and vertical axis as marked by black arrows in Figure 2d, respectively.

A quick screening of the treated sample was carried out using 3D optical microscopy, as shown in Figure 2a. Since the optical microscope was using infinity-corrected optics for reflected light observation, it facilitated the acquisition of long-range line scans, as shown in Figure 2b,c. Figure 2b,c represent long-range line scans along the horizontal and vertical axes, respectively, as shown in Figure 2a. Zoom-in views as marked by a black dashed rectangle in Figure 2b,c were shown in inset (i) and inset (ii) of Figure 2a, respectively. The insets in Figure 2a provided an impression without further details that the treated surface of PC had a microscale roughness. Further details of the surface topography were obtained by the Dektak profilometer. Figure 2d displays the 2D mapping of the treated surface, indicating the step height of the nanostructure as traced by the stylus. Line scans along the white horizontal and vertical lines, as marked in Figure 2d, were shown in Figure 2f,g, respectively. The hills and dips were visible in both line scans. The maximum and minimum step height along the horizontal line scan were found to be ~7 µm and ~−22 µm, respectively. In the case of the line scan along the vertical axis, such hills and dips were found to be ~11 µm and ~−23 µm, respectively. A 3D mapping of the same scan as shown in Figure 2d was acquired and shown in Figure 2e. The islands-like view and the abovementioned height profiles indicated that the surface topography of the treated specimen was indeed of microscopic structures.

**Figure 3:**
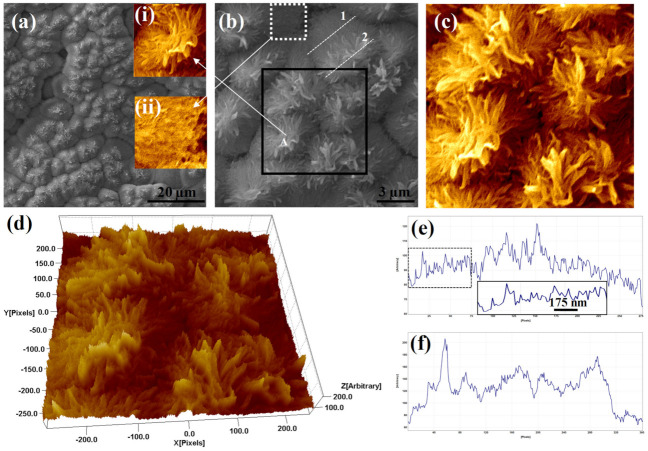
(**a**) FESEM micrograph of treated PC; inset (**i**): an individual flower as marked by the white arrow; inset (**ii**): zoomed-in view of the base nanostructure; (**b**) high-resolution FESEM image indicating several flowers on the top of primary PC nanostructure; (**c**) a zoomed-in view of the selected area as marked by a black square in Figure 3b; (**d**) hawk-eye view of the same area as shown in Figure 3c; (**e**,**f**) line profiles along the white dotted lines marked by “1” and “2” within Figure 3b, respectively.

To validate and reconfirm the inherent characteristics of a hydrophobic surface, one needs to go through nanoscale micrographs similar to those captured by high-resolution FESEM as shown in Figure 3. Figure 3a represents a low-resolution FESEM micrograph confirming fine nanostructures on the top of base nanostructures. A high-resolution FESEM as shown in Figure 3b revealed that the fine nanostructures on the top of base structures were indeed something similar to nanoflowers that consisted of petals of different sizes and shapes. An individual nanoflower as marked by “A” in Figure 3b was shown in the inset of Figure 3a. A further zoomed-in view of a small area as marked by the black square in Figure 3b was displayed in Figure 3c. Seven nanoflowers of different sizes as shown in Figure 3c were observed on the top of base nanostructures. A 3D hawk-eye view of the same area as marked by the black square in Figure 3b was presented in Figure 3d. As mentioned earlier, nanoflowers were observed on the top of base nanostructures. As a result, there were two different layers of nanostructures, one at the base and the other at the top, and thus a combination of these two nanostructures indeed was responsible for making the ultimate surface hydrophobic. A line profile along the white dashed line marked as “1” across the base nanostructure was shown in Figure 3e. A further zoomed-in view of a small section of this line profile as marked by the white dashed rectangle was shown as an inset of Figure 3e. On the other hand, the line profile along the white dashed line marked as “2” across the nanoflower structure was shown in Figure 3f. The sharp hills observed therein corresponded to the petals of the nanoflowers. Hence, it was speculated that such double layers of the nanostructure would provide enough void to satisfy the Cassie and Baxter state and facilitate hydrophobicity of the treated specimen.

**Figure 4:**
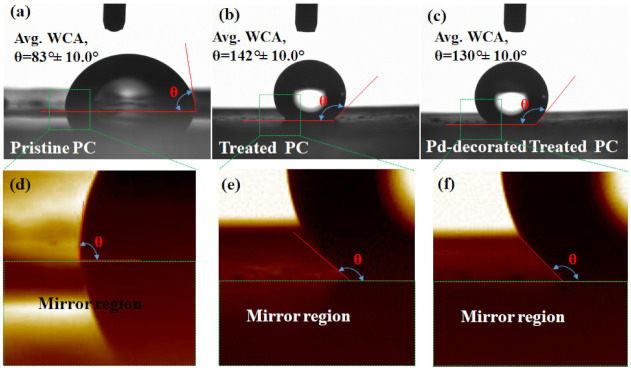
(**a**–**c**) Optical images defining the wetting contact angle of pristine PC, treated PC and Pd-decorated treated PC, respectively; (**d**–**f**) zoomed-in views of the contact site of pristine PC, treated PC and Pd-decorated treated PC, respectively.

It is well-acknowledged that the WCA is the measure of indication of whether the surface is hydrophobic or hydrophilic. Conventionally, a sessile drop test is used to directly measure the contact angle and determine the preferential wetting of the substrate by the reference liquid. Here in this investigation, DI water was used as the reference liquid, and the volume was controlled by an automatic dispenser. Figure 4a–c represents water droplet images of pristine PC, treated PC and Pd-decorated treated PC, respectively. The corresponding average WCA of pristine PC was estimated to be 83 ± 10.0°, as shown in Figure 4a. A further zoomed-in view of the contact region as marked by the green dashed square in Figure 4a is shown in Figure 4d. The lower part of Figure 4d represents the mirror region of the droplet on the pristine PC. It was visible clearly enough, as the pristine PC was noted to be highly transparent. Once the specimen was treated, the sessile drop test confirmed the WCA of the treated PC to be as high as ~142 ± 10.0°, as shown in Figure 4b. As explained earlier, high-resolution FESEM revealed a double layer of nanostructures wherein fine nanoflower-like structures were observed on the top of base nanostructures. Such a combination of nanostructures indeed facilitated enough voids to yield the top surface being hydrophobic. A zoomed-in view of the contact region as marked by the green dashed square in Figure 4b is shown in Figure 4e, reconfirming a high WCA of the same value. The mirror region of the droplet on the treated PC is shown in the lower part of Figure 4e. The mirror image of the treated specimen was found to be blurred, as the transparency of the same specimen dropped substantially. However, the WCA of the Pd-decorated treated PC was still higher compared to that observed in the pristine PC, but it was a bit lower than that observed in the treated PC. Figure 4c displays water droplet images of Pd-decorated treated PC along with the estimated average WCA of ~130 ± 10.0°. A zoomed-in view of the contact region as marked by the green dashed square in Figure 4c is shown in Figure 4f, and the lower part of the same image represents the mirror region of the droplet on the Pd-decorated treated PC.

**Figure 5:**
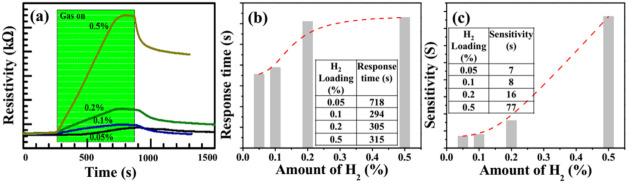
(**a**) Dynamic response of the sensor to different H2 gas concentrations (0.5%, 0.1%, 0.2% and 0.5%) at room temperature; (**b**) response time of the same system; (**c**) sensitivity of the same system.

As explained earlier, the H_2_ sensing measurements were investigated through a special test chamber (Linkam chamber, Model HFS-600E-PB4). This gas chamber was connected to the temperature controller, cooling system and two MFCs using an external power hub supply to introduce an appropriate amount of H_2_. The H_2_ concentration in the Linkam chamber was varied by mixing the test gas (1% H_2_ balanced with N_2_) and dry air. The dynamic response of the Pd-decorated treated PC for H_2_ gas concentrations of 0.05%, 0.1%, 0.2% and 0.5% was shown in Figure 5a. It was observed that for very low concentrations, such as 0.05% of H_2_, the response was delayed and started to show up after ~500 s. For the case of other intakes, such as 0.1%, 0.2|% and 0.5% of H_2_ concentrations, the responses were observed starting from ~300 s, as shown in Figure 5a. However, the response characteristics from each intake of H_2_ concentrations were found to be different. The rising gradients of the dynamic responses for 0.05%, 0.1%, 0.2% and 0.5% of H_2_ concentrations were estimated to be 0.16 (delayed), 0.16, 0.36 and 2.05, respectively.

The response time of the gas sensor is one of the crucial parameters that defines the speed of response of a particular gas sensor. Conventionally, it is measured by the time taken for a sensor to reach 90% of the final indication of saturation. Figure 5b depicts the response time of the specimen in this investigation. It was observed that the intake of H_2_ concentrations of 0.05%, 0.1%, 0.2% and 0.5% took a response time of 718, 294, 305 and 315 s, respectively.

*Sensitivity* is another crucial characteristic that indicates how efficiently the target gas can be detected by the sensor. The sensitivity of a gas sensor is defined as:

(1) $$Sensitivity\;\left(\%\right)=\frac{R_{g}-R_{a}}{R_{a}}\times 100\;$$

where *R_a_* and *R_g_* are the resistance of the sensor in air and target gas, respectively. The electrical resistance of the sensors with and without H_2_ was measured by an Agilent B1500A semiconductor device analyzer (SDA). The sensitivity of the fabricated sensors was calculated within 0.05%–0.5 of H_2_.

Figure 5c depicts the sensitivity of the specimen in this investigation for different intakes of H_2_ concentrations. It was found that the sensitivities for Pd-decorated treated PC at room temperature at 0.05%, 0.1%, 0.2% and 0.5% H_2_ concentrations were 7%, 8%, 16% and 77%, respectively. It is noteworthy that the change in electrical resistance (i.e., sensitivity) and the response time increased with increasing concentrations of H_2_.

## 4. Conclusions

A one-step and low-cost process is one of the prerequisites for an industrial-scale production line. In this context, a simple and inexpensive generic route was reported to achieve polymer-templated hydrophobic nanostructures as H_2_ gas sensing platforms. Double-layer micro and nanostructures of PC were generated that contributed a vital role toward satisfying the Cassie–Baxter state and yielded a hydrophobic surface with a WCA as high as ~142.0 ± 10.0°. Although preliminary topographic investigations through a 3D optical microscope and Dektak profilometer indicated irregular hills and dips of various dimensions that were responsible for creating air bubble pockets therein, an in-depth surface topography was investigated by high-resolution FESEM. Interestingly, it was noticed that fine microscopic flower-like structures of nanoscale petals were populated on the top of base nanostructures. The surface nanostructures were found to be very stable and durable over the course of a year and beyond. Such nano-flowers decorated with Pd-sensing materials exhibited a relatively high WCA and facilitated a high surface area for adsorbing the target gas simultaneously. A sessile drop test confirmed the WCA of the Pd-decorated hydrophobic PC to be as high as ~130.0 ± 10.0°. A lab-built sensing setup was employed to carry out sensing activity of the Pd-decorated hydrophobic PC for H_2_ gas concentrations of 0.05%, 0.1%, 0.2% and 0.5% at room temperature, and the corresponding sensitivity and response time were evaluated.

## Data Availability

The data presented in this study are available on request from the corresponding author.
